# A Precocious Presentation of B-cell Acute Lymphoblastic Leukemia as Infiltrated Plaques on the Face

**DOI:** 10.7759/cureus.4021

**Published:** 2019-02-05

**Authors:** David G Cotter, Brian R Hinds, Charisse Orme

**Affiliations:** 1 Dermatology, Univeristy of California San Diego, San Diego, USA; 2 Dermatology, University of California San Diego, San Diego, USA

**Keywords:** leukemia cutis, acute lymphocytic leukemia, precursor b -cell lymphoblastic lymphoma, acute lymphoblastic leukemia

## Abstract

Leukemia cutis, or infiltration of leukemic cells into the skin, occurs rarely in B-cell acute lymphocytic leukemia (ALL). Herein, we have described a rare, precocious presentation of B-cell ALL presenting as indurated facial plaques in a 69-year-old man. Biopsy of the facial plaques revealed precursor B-cell leukemia/lymphoma in the skin and prompted urgent hematologic-oncologic evaluation. Bone marrow biopsy yielded a final diagnosis of B-cell ALL. The patient underwent induction therapy, and at the last available follow-up, a matched unrelated donor transplant was planned.

## Introduction

Infiltration of the skin by leukemic cells, also known as leukemia cutis, is a rare manifestation of leukemia. While leukemia cutis may be the presenting sign of leukemia, the so-called precocious presentations are rare. Most cases of leukemia cutis occur in association with acute myelogenous leukemia and only rarely arise in association with acute lymphocytic leukemia (ALL) [1-2]. Herein, we have presented a rare case of B-cell ALL presenting as leukemia cutis in a 69-year-old man and discussed the epidemiology of this uncommon disease.

## Case presentation

**Figure 1:**
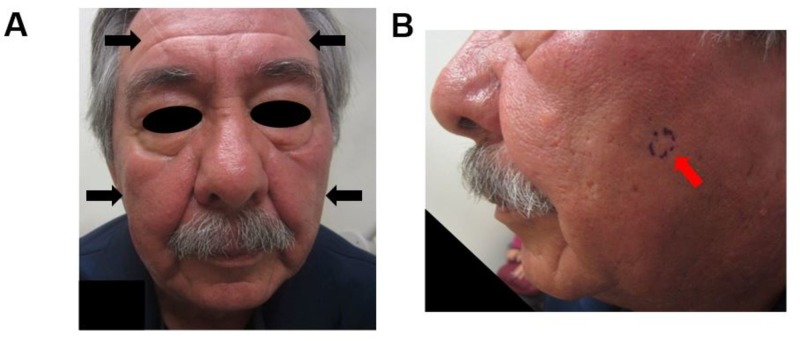
Gross findings Frontal (A) and side view (B) of the patient’s face showing plum-colored, edematous, and infiltrated plaques (indicated by black arrows); biopsy site marked in violet ink and indicated by red arrow (B)

A 69-year-old man with a history of pancytopenia and monoclonal gammopathy of undetermined significance presented with a chief complaint of "the skin on my face and scalp feels uncomfortable". He complained of diffuse facial fullness and burning of approximately three-week duration. Physical examination demonstrated facial edema with firm, violaceous papules subtly coalescing into infiltrative plaques (Figures 1A-1B).

**Figure 2:**
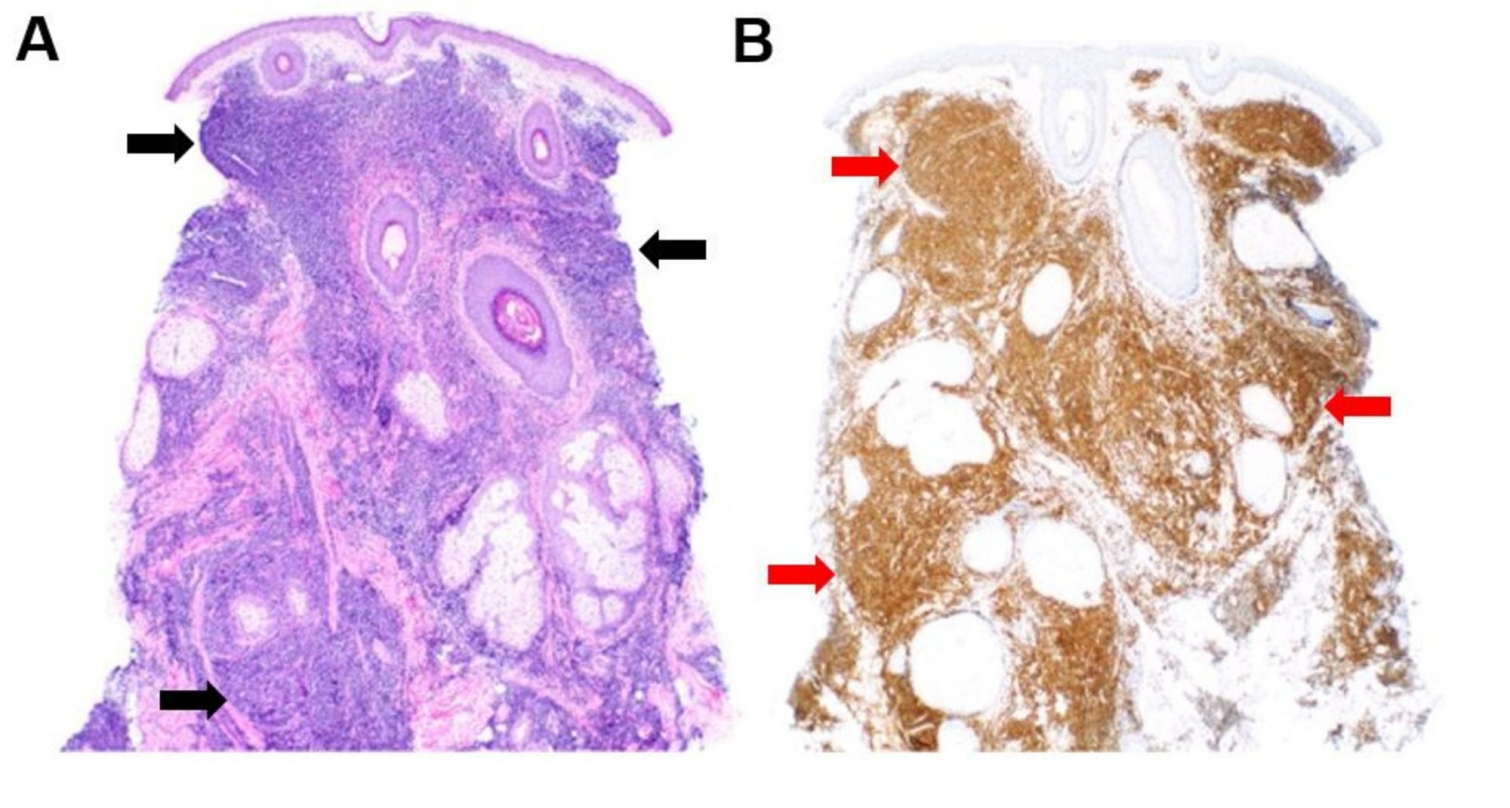
Microscopic findings Skin biopsy from the patient’s left cheek stained with hematoxylin and eosin highlights a pan-dermal lymphoid infiltrate, indicated by the black arrows (original magnification 40x; A). Paired staining of the patient's skin biopsy with Pax-5 (brown staining indicated by the red arrows) indicates B cell lineage among the cells of interest (original magnification 40x; B)

Punch biopsy obtained from the left cheek revealed a pan-dermal lymphoid infiltrate comprising monomorphous, medium to large lymphocytes with high-grade nuclear atypia (Figure 2A – hematoxylin-eosin, 40x magnification). Immunoperoxidase staining was performed to query the differential diagnosis, which ultimately included precursor B-cell leukemia/lymphoma, myelogenous leukemia cutis, blastic mantle cell lymphoma, and natural killer (NK)/T-cell lymphoma. The atypical lymphocytes lacked cluster of differentiation (CD) 3 or CD56 expression, excluding cutaneous T-cell lymphoma and NK/T-cell lymphoma, respectively. Myeloperoxidase staining was negative, thus excluding myelogenous leukemia. By contrast, the tumor cells strongly expressed Paired Box 5 (Pax-5), as shown in Figure 2B (Pax-5 staining, 40x magnification), and displayed partial positivity for CD79a. Based on these findings, a high-grade B-cell neoplasm was suspected, and subsequent staining for terminal deoxynucleotidyl transferase (TdT) immunostaining revealed avid immunopositivity. The histomorphology and immunophenotype (Pax-5+, CD79a+/-, and TdT+) substantiated a diagnosis of precursor B-cell leukemia/lymphoma in the skin, pending a systemic workup.

Hematologic-oncologic evaluation was initiated. The patient was found to have a profound leukocytosis (43.4 x 10^3^/microliter). Approximately 40% of these cells were atypical myelocytes of uncertain lineage. A bone marrow biopsy showed hypercellular marrow with 94% blast cells. Marrow blast cells expressed Pax-5, TdT, CD19, and CD79a, and were variably positive for CD20, ultimately confirming a diagnosis of acute B-cell lymphoblastic leukemia. The patient was admitted for induction therapy with cyclophosphamide, vincristine, adriamycin, dexamethasone, methotrexate, and cytarabine (Hyper CVAD A). At the last available follow-up, human leukocyte antigen typing was pending in anticipation of a matched unrelated donor transplant.

## Discussion

ALL is an extraordinarily rare malignancy. In 2018, an estimated 5,960 cases of ALL were diagnosed, accounting for only 0.3% of all new cancer cases in the United States [3-4]. Furthermore, leukemia cutis rarely occurs in this disease [2,5-6]. Leukemia cutis most commonly occurs in association with acute myelogenous leukemia, in which it occurs in less than 4% of cases [1]. Moreover, B-cell ALL occurs most frequently in pediatric populations. Only around 10% of all cases of ALL occur in patients over the age of 65 [3]. The five-year survival of B-cell ALL in children is as high as 90% [3] but sadly falls to less than 15% in patients aged 60-70 years [7-8]. While the prognosis of ALL is multifactorial, enhanced pediatric survival rates are largely attributable to genetic features such as hyperdiploidy and translocation (12;21), and comparably fewer comorbidities in children [9-10]. 

Overall, only 15 patients with leukemia cutis secondary to B-cell ALL have been reported in the literature [2]. Similarly, there are less than 20 reported patients with primary cutaneous precursor B-cell lymphoblastic lymphoma [5-6]. These two disease entities differ based on their degree of systemic involvement. Leukemia cutis due to B-cell ALL invariably represents the cutaneous involvement of leukemic bone marrow-derived cells. In contradistinction, primary cutaneous precursor B-cell lymphoblastic lymphomas occur at extranodal sites, frequently manifesting in the skin and bone. Despite this ontogenetic distinction, both diseases are treated in parallel fashion with aggressive chemotherapy regimens designed for ALL [5]. Among cases of leukemia cutis due to B-cell ALL, approximately 1/3 of cases occur in pediatric patients, 1/5 of cases occur in patients over 65, and 1/3 of cases occur during relapse of B-cell ALL [2]. Thus, the patient presented herein represents a rare manifestation of a rare disease and highlights the importance of clinical-pathologic correlation.

## Conclusions

Leukemia cutis represents an infrequent cutaneous manifestation of hematologic malignancies. While it occurs in 3% to 4% of patients with acute myelogenous leukemia, it occurs much less frequently in association with ALL. Only 20 patients with leukemia cutis due to B-cell ALL have been reported and many of these occurred in pediatric patients or in association with relapsed ALL. Our adult patient did not have a preceding diagnosis of leukemia prior to his biopsy. Thus, immunohistochemical staining was paramount in identifying the cellular lineage of the malignant infiltrate. In our patient, we were able to exclude myeloid, T-cell, and NK/T-cell lineage because the malignant cells did not express the myeloid marker myeloperoxidase, the T-cell marker CD3, or the NK/T-cell marker CD56. By contrast, our patient’s malignant infiltrate stained for the B-cell markers Pax5, TdT, and CD79a, indicating B-cell lineage. In our patient, skin biopsy prompted life-altering hematologic oncologic evaluation. Thus, this case exemplifies the importance of clinical-pathologic correlation.
